# Esomeprazole-Induced Chest Pain: A Case of an Unexpected Serious Adverse Reaction to a Proton Pump Inhibitor

**DOI:** 10.1155/2020/5693545

**Published:** 2020-05-14

**Authors:** Melanija Ražov Radas, Aleksandar Knežević, Vladimir Trkulja

**Affiliations:** ^1^Department of Gastroenterology, Zadar General Hospital, Zadar, Croatia; ^2^Department of Cardiology, Zadar General Hospital, Zadar, Croatia; ^3^Department of Pharmacology, Zagreb University School of Medicine, Zagreb, Croatia

## 1. Introduction

Considering the high prevalence and chronic nature of the acid peptic disease, PPIs have been extensively used and their safety has attracted much attention. Esomeprazole has well-established efficacy and safety in treatment of all clinical manifestations of the acid peptic disease. As a group, PPIs inherently bear a potential for drug-drug interactions reflecting either on disposition of PPIs (e.g., their dependence on CYP-mediated metabolism, pantoprazole excepted) or on disposition of concomitant drugs (e.g., reduced gastrointestinal acidity may affect absorption of certain drugs; apart from pantoprazole, PPIs may inhibit CYP2C19). Clinically relevant drug-drug interactions with esomeprazole (or PPIs) have been only sporadic, likely also due to the awareness about this possibility. It is important to note that warnings have been issued regarding the potential of PPIs (including esomeprazole, but likely excluding pantoprazole) to reduce the efficacy of clopidogrel (inhibition of clopidogrel activation by CYP2C19), but this is not the subject of our presentation (our patient did not take clopidogrel). Also, the association between long-term use of PPIs and an increased risk of fractures has been recognized. Esomeprazole, as well as all marketed PPIs, has an overall excellent safety profile reflected by the fact that most of them have a world-wide over-the-counter status. In particular, cardiovascular safety of esomeprazole and omeprazole appears good and they seem not to increase the risk of adverse cardiac events. We present a patient with a condition that appears to be a case of esomeprazole-induced chest pain with changes that we recorded with the standard 12-lead electrocardiogram (ECG) which should be related to coronary ischemia but not typical for it.

## 2. Case Report

We present a patient with a condition that appears to be a case of esomeprazole-induced chest pain with ECG changes indicative of myocardial ischemia, albeit not typical for it.

*Visit 1*. In November 2004, a 57-year-old woman, nonsmoker with a 10-year history of hypertension (managed by atenolol 50 mg/day and aspirin 100 mg/day), presented with noncharacteristic chest pressure, palpitations, and shortness of breath during physical activity. Her sitting blood pressure (BP) was 140/90 mmHg, and physical examination, chest X-ray, and routine laboratory tests were unremarkable. Standard 12-lead electrocardiogram showed a sinus rhythm with 55 bpm, normal electrical axis, and shallow negative T-waves in V1–V3 leads, indicating possible myocardial ischemia. Echocardiography findings were normal. The exercise test showed a hypertensive reaction to strain, no rhythm disturbance, normal functional capacity, and a negative test of coronary reserve. However, since a negative coronary reserve test is possible even with a coronary vessel disease, coronarography was indicated that showed normal epicardial coronary vessels. Her difficulties were considered as a possible anginal discomfort. She was prescribed nitroglycerin spray to be used as needed in the case of remitting difficulties. In October 2006, in order to improve her BP control, antihypertensive treatment was changed to bisoprolol 5 mg/day, perindopril 2 × 4 mg/day, and aspirin 100 mg/day.

*Visit 2*. On May 28, 2007, she reported new complaints. Three weeks earlier, she had been diagnosed with gastroesophageal reflux disease (GERD) and started treatment with oral esomeprazole 20 mg/day. Since then, three to four hours after esomeprazole consumption (coinciding with “post-peak” esomeprazole concentrations [1]), she would feel chest constriction identical to anginal difficulties, which would stop after administration of nitroglycerin. Her sitting BP was 120/80 mmHg, and her physical examination, routine laboratory tests, and a 12-lead ECG (Fogure 1(a)) were unremarkable. She was advised to continue her treatment and to keep records of anginal difficulties.

*Visit 3*. Nineteen days later, on June 16, 2007, esomeprazole was withdrawn since the difficulties related to its consumption persisted.

*Visit 4*. On September 4, 2007, she reported no anginal difficulties since withdrawal of esomeprazole three months earlier. However, her GERD difficulties are prominent. Her BP is 140/95 mmHg, and she is clinically unremarkable. After a consultation with a clinical pharmacologist, 20 mg of esomeprazole is administered. She is observed for a day but only complains about a slight headache. She is advised to take esomeprazole the next morning and to refer to the cardiology unit for observation.

**Figure 1:**
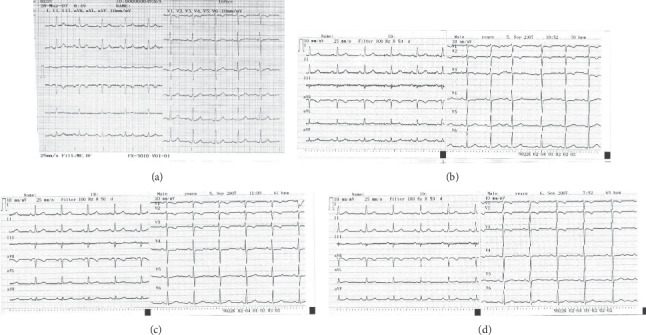
Standard 12-lead ECG recordings: (a) On May 28, 2007, no actual difficulties; (b) at 10 : 52 a.m., on September 5, 2007, before the chest pain episode; (c) at 11 : 09 a.m., on September 5, 2007, during the chest pain episode; (d) on September 6, 2007, at 7 : 52 a.m., no difficulties. Note that patient's sex was erroneously recorded as “male.”

*Visit 5*. On September 5, 2007, she took 20 mg of esomeprazole at 6 a.m. At 10 a.m., her BP is 135/90 mmHg, and she is clinically unremarkable as is a 12-lead ECG taken at 10 : 52 a.m. (Figure 1(b)). However, at 11 : 05 a.m., she started feeling anginal discomfort. At that time (11 : 09 a.m.), ECG showed shallow negative T-waves with a milder ST-segment depression (by around 1 mm) in V1 – V4 leads (Figure 1(c)). The difficulties resolved a few minutes after administration of two sprays of nitroglycerin. Her BP measured on several occasions between 10 : 00 and 11 : 30 a.m. did not change. The next morning, she took no esomeprazole. Her ECG was unremarkable (Figure 1(d)).

## 3. Discussion

Esomeprazole is a potent selective inhibitor of the H^+^/K^+^ ATPase in the parietal cells of the gastric mucosa, i.e., a proton pump inhibitor. It is a pure *S*-enantiomer of omeprazole, a racemic mixture, the first marketed and prototypical PPI. Just as its ancestor, esomeprazole has well-established efficacy and safety in treatment of all clinical manifestations of the acid peptic disease [2]. Clearance of esomeprazole is apparently less dependent on CYP2C19 than that of omeprazole [3]. Proton pump inhibitors do not belong among known causes of ischemic or nonischemic chest pain [4]. But, it is important to note that warnings have been issued regarding the potential of PPIs (including esomeprazole, but likely excluding pantoprazole) to reduce the efficacy of clopidogrel (inhibition of clopidogrel activation by CYP2C19) [5]. A thorough evaluation of omeprazole and esomeprazole by FDA in 2007 concluded no association between the use of these two drugs and adverse cardiac events [6], and chest pain, as a possible side effect, is not part of the label of esomeprazole [1].

The mechanism(s) underlying the present observation remain(s) elusive: BP measurements did not indicate precipitation of chest pain by low or high BP; neither bisoprolol nor perindopril are known to affect disposition of esomeprazole and *vice versa*; esomeprazole is not known to affect disposition of bisoprolol or perindopril [2, 3]. PPIs are not among the known causes of ischemic or nonischemic chest pain [7]. The current dechallenge and rechallenge data strongly suggest a causal relationship between esomeprazole and the observed difficulties although the underlying mechanism(s) remain(s) elusive particularly since interactions between esomeprazole and coadministered treatments are not known [6]. Physical examination, of our patient, chest X-ray, and routine laboratory tests were unremarkable. Standard 12- lead electrocardiogram showed shallow negative T-waves in V1–V3 leads, indicating possible left atrial enlargement. Echocardiography findings were normal. The exercise test showed a hypertensive reaction. At the time, we were unable to determine troponin values. Coronarography was indicated and showed normal epicardial coronary vessels. Because the patient had ECG changes typical of transient myocardial ischemia and without myocardial damage, we characterized the reaction as microvascular dysfunction.

## 4. Conclusion

Esomeprazole was withdrawn permanently. Her GERD treatment continued with pantoprazole. Her antihypertensive treatment was not changed. Her BP has been successfully managed, and she has had no anginal discomfort since. This adverse reaction was reported to the Croatian agency for medicines and medical devices. In accordance with the criteria of the Council for International Organizations of Medical Sciences, it was judged as serious. Based on the rechallenge and dechallenge effects, the Croatian Agency judged its causal relationship to esomeprazole as certain.

Hence, the reported adverse reaction fulfills criteria of an unexpected or type B (atypical and idiosyncratic) adverse reaction.

## Data Availability

The data used to support the findings of this study are available from the corresponding author upon request.
